# Index Coded Automatic Repeat Request (ARQ)

**DOI:** 10.3390/e22080869

**Published:** 2020-08-07

**Authors:** Sang Won Choi

**Affiliations:** Train Control & Communication Research Team, Korea Railroad Research Institute (KRRI), Uiwang-si 16105, Korea; swchoi@krri.re.kr; Tel.: +82-31-460-5668

**Keywords:** Automatic Repeat Request (ARQ), index coding, random linear network coding

## Abstract

In this paper, an index-coded Automatic Repeat Request (ARQ) is studied in the perspectives of transmission efficiency and memory overhead. Motivated by reducing significant computational complexity from huge matrix inverse computation of random linear network coding, a near-to-optimal broadcasting scheme, called index-coded Automatic Repeat Request (ARQ) is proposed. The main idea is to consider the principal packet error pattern across all receivers. With the help of coded side information formed by successfully decoded packets associated with the dominant packet error pattern, it is shown that two contradictory performance metrics such as transmission efficiency and transmit (receive) cache memory size for index coding (decoding) can be enhanced with a reasonable trade-off. Specifically, the transmission efficiency of the proposed scheme is proved to be asymptotically optimal, and memory overhead is shown to be asymptotically close to the conventional ARQ scheme. Numerical results also validate the proposed scheme in the sense of memory overhead and transmission efficiency in comparison with the conventional ARQ scheme and the optimal scheme using random linear network coding.

## 1. Introduction

From an information-theoretic point of view, a information can decrease uncertainty of the corresponding information, which increases the source coding rate [1]. The side information can be used to optimize rate regions in multi-user channels. One of typical examples is the multiple access channel whose achievable scheme is multi-user detection [2] by leveraging side information from detecting other users’ signals. On the other hand, index coding is a transmission technique that exploits the side information known a priori at each receiver [3,4,5,6]. With the help of the side information, the number of retransmissions can be significantly decreased in multicasting and broadcasting scenarios. In general, network coding [7] includes index coding as a special case, which contributes to efficient transmission by diminishing the number of transmissions in wired communications. Essentially, index coding and network coding are lying on the same line in the sense that those two coding schemes utilize coded information as a linear combination of independent information packets. Interestingly, the equivalence of two coding schemes has been shown by proving the fact that index coding is a special case of network coding and vice versa [6]. Recently, the optimal binary index code length has been characterized based on the minimum rank of the corresponding design matrix when each receiver has coded side information, which is more efficient in the sense of memory overhead in comparison with using naive side information [8]. Basically, index coding or network coding is assumed to utilize feasible side information at the transmitter. In broadcast channels, such an assumption is valid by using feedback from each receiver. Here, the feedback from the receivers is usually assumed to be error free. Motivated by the effectiveness in retransmission via coded data transmission, coding schemes and their analysis for broadcasting or multicasting have been studied. Consequently, a number of transmission protocols have been proposed showing that transmission efficiency is enhanced beyond the conventional broadcasting systems [9,10,11,12,13,14,15]. The main contribution of this paper is to investigate an efficient broadcasting scheme in both finite and asymptotic performance perspectives. Specifically, index-coded Automatic Repeat reQuest (ARQ) is proposed based on statistical property across all the receiver’s packet error pattern. Furthermore, asymptotic optimality of the proposed scheme is shown in terms of transmission efficiency according to feasible ranges of packet error probability and the number of users. In addition, the proposed scheme is validated in terms of memory overhead and transmission efficiency. The rest of this paper is organized as follows. In Section 2, a system model is described, and the proposed ARQ schemes are analyzed in Section 3 with some motivating examples. Then, the index-coded ARQ with low complexity is also proposed with performance analysis in Section 4, which is validated through numerical evaluations in Section 5. Finally, the paper is concluded in Section 6.

## 2. System Model

A wireless broadcasting system is considered, which is composed of one transmitter (Tx) and *K* receivers (Rxs). In the broadcasting system, packet transmission is assumed, where each packet is encoded using channel encoding at the physical layer so that each packet becomes robust to various noise sources caused by the low noise amplifier, fading, and multi user interference in wireless channels. However, there can always be packet error events so that ARQ is usually used to make packet transmission reliable at the cost of increased latency. Here, it is assumed that the packet error probabilities $P_{k}$’s at the *k*-th Rx for k∈{1,2,⋯,K} are all equal to *P* at any time instance. The packet error probabilities $P_{k}$’s are different in general. Note that the proposed coding scheme can be applied to the general case without any change of the coding scheme. Since the essence behind the coding scheme is the same regardless of the assumption of the packet error probability, this paper focuses on the homogeneous Rx environment, that is, $P_{k}=P$ for k∈{1,2,⋯,K}. Assuming that an ARQ scheme is used for a delay tolerant service (e.g., file transmission), we have the following transmission phases:

- The first phase: Transmission of original *N* data packets
- The *n*-th phase: Retransmission of erroneous packets in the (n−1)-th phase

According to ACK or NACK feedback sent from each Rx for the *N* packets transmitted in the first phase, the Tx can classify the *N* packets into multiple disjoint predetermined sets.

Specifically, $M^{\left[i\right]}$ for i=1,2,⋯,K is defined as the set of packets, where *i* Rxs among *K* Rxs decode the packets successfully and send ACK feedback. The set $M^{\left[i\right]}$ has disjoint subsets $M_{j}^{\left[i\right]}$’s according to the corresponding ACK combination over *K* Rxs, where $j=0,\;1,\;\cdots ,{\;}_{K}C_{i}$. Here, the set $M^{\left[i\right]}$ will be called as the *i*-th packet error pattern set.

We define $m_{j}^{\left[i\right]}$ as a packet in the subset $M_{j}^{\left[i\right]}$. Table 1 and Table 2 illustrate all $M^{\left[i\right]}$’s and $M_{j}^{\left[i\right]}$’s when K=2 and K=3 (Note that given error pattern index *i*, the number of packets in each of $M_{j}^{\left[i\right]}$ can be arbitrarily close to $N\cdot_{K}C_{i}/\left(\sum_{i=0}^{K}{}_{K}C_{i}\right)$ assuming that *N* is sufficiently large based on ergodic theorem [16]).

In this paper, an efficient coding scheme is proposed for broadcasting systems assuming delay tolerant data services when *N* packets are transmitted for sufficiently large *N*. Motivation for using the ergodicity comes from the fact that the stochastic properties can be leveraged when *N* is large enough. As a performance metric, transmission efficiency η is defined as

(1) $$\begin{array}{c} \eta =\frac{M}{N}, \end{array}$$

where *M* is the total number of transmitted packets including retransmission. Thus, the optimal scheme aims to minimize the number M−N, indicating number of retransmitted packets.

## 3. Motivating Examples When K=2 and K=3

In this Section, naive index-coded ARQ schemes when K=2 and K=3 have been considered by starting with the following lemmas.

**Lemma** **1.** *For independent N packet transmissions with a packet error probability of P, the average number of required total transmissions for successful reception of N packets can be arbitrarily close to N/(1−P) with a probability of 1−ϵ for any ϵ>0. Here, N is assumed to be a sufficiently large nonnegative integer.*

**Proof of Lemma** **1.** It is straightforwardly proved using strong typicality [17] and is omitted.  □

**Lemma** **2.** *When N goes to infinity, the optimal transmission efficiency of 1/(1−P) is achievable by random linear network coding.*

**Proof of Lemma** **2.** It essentially follows from Reference [18].  □

**Remark** **1.** *For achieving the optimal transmission efficiency, random linear network coding [18] requires high computational complexity for inverse operation of a matrix with size of N×N, which ranges from $O(N^{2.373})$ to $O(N^{3})$ depending on the applied numerical algorithms. Thus, when N is sufficiently large, a significant challenge on complexity is confronted in practice.*

### 3.1. When K=2

In case of utilizing index coding over multiple packets with K=2, transmission efficiency can be enhanced at the cost of storing additional coded side information. The coding scheme is described as follows:

- For the zero-th packet error pattern set, no index coding is feasible since there is no packet that is decoded successfully. Thus, the average number of retransmitted packets approaches
  (2) $$\frac{NP^{2}}{{\left(1-P\right)}^{2}}$$
  for sufficiently large *N*.
- For the 1-st packet error pattern set, the index coding can be utilized over two different packets for broadcasting in the two packet error pattern subsets of $M_{1}^{\left[1\right]}$ and $M_{2}^{\left[1\right]}$. When $m_{j}^{\left[i\right]}$ denotes a packet in the subset of $M_{j}^{\left[i\right]}$, the index-coded packet is given by
  (3) $$m_{1}^{\left[1\right]}⊕m_{2}^{\left[1\right]},$$
  where ⊕ represents the XOR operation. Here, the index coding is effectively used because each Rx has a successfully decoded packet in $M_{1}^{\left[1\right]}$ or $M_{2}^{\left[1\right]}$.
- For the second packet error pattern set, there is no packet error event, resulting in no retransmission.

Finally, the total number of transmitted packets converges to

(4) $$\frac{N(1-P+P^{2})}{{\left(1-P\right)}^{2}}$$

on average for the original *N* broadcasted packets, where *N* is sufficiently large.

### 3.2. When K=3

When an index-coded ARQ is used for broadcasting with K=3, the design of a more sophisticated coding scheme is required, which is as follows:

- For the zero-th packet errror pattern set, there is no side information from successfully decoded packets. Hence, the average number of retransmitted packets is
  (5) $$\frac{NP^{3}}{{\left(1-P\right)}^{3}}$$
  for sufficiently large *N*.
- For the first packet error pattern set, each Rx decodes packets only in one of packet error pattern subsets of $M_{1}^{\left[1\right]}$, $M_{2}^{\left[1\right]}$, and $M_{3}^{\left[1\right]}$. Thus, based on the side information, the following index coding can be used at the Tx:
  (6) $$m_{1}^{\left[1\right]}⊕m_{2}^{\left[1\right]},\;m_{1}^{\left[1\right]}⊕m_{3}^{\left[1\right]},\;m_{2}^{\left[1\right]}⊕m_{3}^{\left[1\right]},$$
  where $m_{j}^{\left[i\right]}$ is a packet in the jth subset in the ith packet error pattern set. At the Rx side, three packets $m_{j}^{\left[i\right]}$’s with j∈{1,2,3} can be decoded successfully if more than or equal to 2 index-coded packets in Equation (6) is feasible. Consequently, the total number of retransmitted packets converges to
  (7) $$\frac{3N{\left(1-P\right)}^{2}P}{3\left(1-P^{2}\right)P+{\left(1-P\right)}^{3}},$$
  asymptotically.
- For the second packet error pattern set, each Rx can use two successfully decoded packets in packet error pattern subsets of $M_{1}^{\left[2\right]}$, $M_{2}^{\left[2\right]}$, and $M_{3}^{\left[2\right]}$ so that the following index coding is applied:
  (8) $$m_{1}^{\left[2\right]}⊕m_{2}^{\left[2\right]}⊕m_{3}^{\left[2\right]}.$$
  In this case, each Rx can decode packet if index-coded packet belongs to packet error pattern subsets of $M_{1}^{\left[2\right]}$, $M_{2}^{\left[2\right]}$, and $M_{3}^{\left[2\right]}$ and the index-coded packet in (8) is decoded successfully. Thus, the total number of retransmission packets converges to
  (9) $$\frac{N{\left(1-P\right)}^{2}P}{{\left(1-P\right)}^{3}}$$
  on average.
- For the third packet error pattern set, all packets are decoded successfully at each Rx. Thus, the total number of retransmitted packets is zero.

Finally, the the total number of transmitted packets converges to

(10) $$\frac{N(5P^{4}-3P^{3}+1)}{{\left(1-P\right)}^{3}\left(2P+1\right)}$$

asymptotically for large *N*.

Table 3 depicts the transmission efficiency of the naive index-coded ARQ for broadcasting when K=2 and K=3 for various packet error probabilities. It is observed that index-coded ARQ exhibits nearly optimal transmission efficiency for a wide range of packet error probabilities.

## 4. Disadvantages of Conventional ARQ and Naive Index Coded ARQ

In this Section, disadvantages of the conventional ARQ and naive index-coded ARQ schemes are analyzed in the perspective of transmission efficiency and memory overhead for practical use.

### 4.1. Conventional ARQ with No Index Coding: Inefficiency in the Sense of Total Number of Transmission

For the conventional ARQ without the index coding, packet retransmission is activated whenever at least one Rx fails to decode the desired packet (In this paper, a broadcasting system is considered, where the desired packets of each Rx are assumed to be all *N* packets). Thus, when *N* goes to infinity, the average number of total transmission packets can be arbitrarily close to

(11) $$\frac{N}{{\left(1-P\right)}^{K}}$$

from Lemma 1. Consequently, the average number of total packet transmission is increased exponentially with *K*, which may result in inefficient resource usage. Moreover, one Tx should store the entirely transmitted *N* packets for retransmission.

### 4.2. Na$\ddot{i}$ve Index-Coded ARQ: Inefficiency in the Sense of Memory overhead

#### 4.2.1. Memory Overhead When K=2 and K=3

From the coding scheme when K=2 and K=3 in the previous Section, additional memory overhead for the packets to be stored converges to

(12) N{1+(1−P)P}

and

(13) $$N\left\{1+3\left(1-P\right)P^{2}+{\left(1-P\right)}^{2}P\right\}=1+P+P^{2}-2P^{3},$$

respectively when *N* is sufficiently large.

#### 4.2.2. Memory Overhead for the General Case

We consider how many packets are to be stored to utilize index coding. When the number of users are *K*, the number of packet error pattern is K+1. We define *t* as a specific packet error pattern index. Then, *t* ranges from 0 to K.

For the *t*-th packet error pattern, total number of packet error pattern subsets are given by

(14) $$\begin{array}{c} {}_{k}C_{t}=\frac{k!}{(k-t)!t!}, \end{array}$$

where is the product of all positive integers less than or equal to k for a positive *k* with 0!=1. For any packet in the ${}_{k}C_{t}$ packet error pattern subsets, the number of packet error across *K* Rxs is equal to *t*. Thus, for each Rx, the number of successfully decoded packets becomes $t{(}_{k}C_{t})/K$. This is because equal number of known packets are allocated for each packet error pattern from equal error probability over *K* Rxs.

Finally, packets to be stored for index coding is given by

(15) $$N\{{}_{{}_{K}C_{t}}C_{\left(\frac{t\cdot_{K}C_{t}}{K}+1\right)}\}\cdot {\left(1-P\right)}^{t}\cdot P^{K-t}$$

asymptotically for the packet error pattern index *t* which is a nonnegative integer *t* with 0≤t≤K−1. Note that when t=K, there is no packet error for all Rxs so that memory for index coding is not required.

Basically, memory overhead becomes significant when *K* increases and all packet combinations for the index coding is used. Motivated by the fact that a packet error pattern with t=K−1 is the most dominant, the index coding over the packets in the dominant packet error pattern set is considered with decreased memory overhead. From the generalized memory overhead in Equation (15), the memory overhead when t=K−1 is given by

(16) $$N{\left(1-P\right)}^{K-1}P,$$

which is approximated as

(17) N{1−(K−1)P}P

assuming that *P* goes to 0. Therefore, the additional memory overhead Equation (17) is asymptotically negligible when $P\leq O(K^{-(1+\epsilon )})$ with sufficiently large *K* and ϵ>0. Here, *O* represents Landau’s symbol used in complexity theory.

In comparison with the conventional ARQ scheme, the proposed scheme has smaller memory overhead at the transmitter by using side information. Specifically, in case of the conventional ARQ scheme, the number of packets to be stored is given by

(18) $$N\left\{1-{\left(1-P\right)}^{K}\right\}$$

at the transmitter. For the proposed scheme, the packets in the packet error pattern set $M^{\left[K-1\right]}$ can be stored in the form of coded side information, which is feasible by the fact that K−1 receivers decode effectively by using the successfully decoded packets.

## 5. Proposed Scheme: Simplified Index-Coded ARQ

In this Section, the memory overhead issue is dealt with in the sense of storing additional packets for leveraging an index coding. Considering the feasible overhead in a practical sense, retransmission coding scheme with index coding is proposed by utilizing only dominant coded side information, and show the asymptotic optimality of the proposed index coding scheme in the sense of the transmission efficiency.

### 5.1. Coding Scheme

Assuming the memory overhead in Equation (16), the following coding scheme can be feasible. Here, it is assumed that *N* packets are already broadcasted to all Rxs, and all Rxs have already transmitted ACK or NACK for each received packet to the Tx.

According to packet error pattern and transmission phase, coding scheme is determined at Tx side. Specifically,

- The 1-st phase: Transmission of original *N* packets is performed.
- From the 2-nd phase: If packets belonged in the packet error pattern set and its subsets of $M^{\left[K-1\right]}$ and $M_{j}^{\left[K-1\right]}$, the following index coding is accomplished.
  (19) $$m_{1}^{\left[K-1\right]}⊕m_{2}^{\left[K-1\right]}⊕\cdots ⊕m_{K}^{\left[K-1\right]}.$$
  Else, naive retransmission of corresponding packets is done. It is worthy noting that the packet error pattern is determined by the ACK and NACK pattern after the 1-st packet transmission.

On the other hand, the coding scheme at the receiver side is as follows: Since the dominant coded side information Equation (19) is used for the receiver, it is sufficient to explain the decoding rule for the packets belonged in the packet error pattern set and its subset, that is, $M^{\left[K-1\right]}$ and $M_{j}^{\left[K-1\right]}$’s. Specifically, the packets in $M_{j}^{\left[K-1\right]}$’s is decoded by using stored coded side information, which is given by Equation (20). In Equation (20), $\left\{m_{1}^{\left[K-1\right]}⊕m_{2}^{\left[K-1\right]}\cdots m_{K}^{\left[K-1\right]}\right\}$ means coded side information stored at the receiver for decoding, and $\left\{m_{1}^{\left[K-1\right]}\cdots ⊕m_{j-1}^{\left[K-1\right]}⊕m_{j+1}^{\left[K-1\right]}\cdots m_{K}^{\left[K-1\right]}\right\}$ indicates the index-coded packet at the transmitter.

(20) $$\{m_{1}^{\left[K-1\right]}⊕m_{2}^{\left[K-1\right]}\cdots m_{K}^{\left[K-1\right]}\}⊕\{m_{1}^{\left[K-1\right]}\cdots ⊕m_{j-1}^{\left[K-1\right]}⊕m_{j+1}^{\left[K-1\right]}\cdots m_{K}^{\left[K-1\right]}\}=m_{j}^{\left[K-1\right]}.$$

### 5.2. Asymptotic Optimality in the Sense of the Transmission Efficiency

Basically, index-coded packet transmission guarantees efficient broadcast transmission by effective use of side information stored at each Rx. The proposed scheme considers most dominant packet error event given P ranging from 0 and 1. In a strict sense, *P* is always greater than or equal to 1/2.

For total number *N* of broadcasted packets, the number of transmitted packets including retransmission becomes

(21) $$\begin{array}{c} \{\frac{1}{{\left(1-P\right)}^{K}}-\frac{(K-1)P}{1-P}\}, \end{array}$$

which is derived by using packet error event for the (K−1)-th packet error pattern and Lemma 1.

When *K* goes to infinity and $P<O(K^{-(1+\epsilon )})$ with ϵ>0, Equation (21) is approximated as

(22) $$\begin{array}{c} \frac{N}{1-P}\{\frac{1}{1-(K-1)P}-\left(K-1\right)P\}, \end{array}$$

which converges to

(23) $$\begin{array}{c} \frac{N}{1-P}. \end{array}$$

Note that Equation (23) corresponds to the optimal transmission efficiency using random linear network coding [18]. Consequently, the proposed scheme is shown to be asymptotically optimal in the sense of transmission efficiency.

### 5.3. Reduction of Memory Overhead

Basically, the proposed scheme utilizes index coding over packets belonged in a specific error pattern index set and its subsets, that is, $M^{\left[K-1\right]}$ and $M_{j}^{\left[K-1\right]}$’s. Therefore, the amount of reduced memory overhead in comparison with that of the nave index-coded ARQ is given by

(24) $$\begin{array}{c} \sum_{t=0}^{K-2}N\{{}_{{}_{K}C_{t}}C_{\left(\frac{t\cdot_{K}C_{t}}{K}+1\right)}\}\cdot {\left(1-P\right)}^{t}\cdot P^{K-t}, \end{array}$$

from Equations (15) and (16). It is worthy noting that the coded side information [8] can be effectively leveraged at the receiver, which diminish additional memory overhead. In other words, it is sufficient to store corresponding index-coded packets belonged in specific error pattern index set and its subsets.

## 6. Numerical Results

In the perspective of asymptotic performance, the proposed scheme was shown to achieve asymptotically optimal transmission efficiency along with negligible (additional) memory overhead when $P<O(K^{-(1+\epsilon )})$ (Note that the performance improvements are still guaranteed even when the packet error probabilities are different for all users. This is because the proposed scheme can still utilize side information stored at each Rx, where the number of stored packets is given by Equaition (18)). In this Section, finite performance of the proposed scheme is evaluated and validated in comparison with optimal scheme using random linear network coding and conventional ARQ.

### 6.1. Transmission Efficiency

In the sense of transmission efficiency, random linear network coding can achieve reliable N packet broadcasting to *K* Rxs asymptotically without packet error by transmitting N/(1−P) at the cost of the high computational complexity for calculating inverse of N×N matrix. Here, the random linear network coding scheme is referred to as the optimal scheme. On the other hand, the proposed scheme considers use of coded side information based on successfully decoded packets across all Rxs, which can diminish the retransmission time from the Tx.

**Figure 1:**
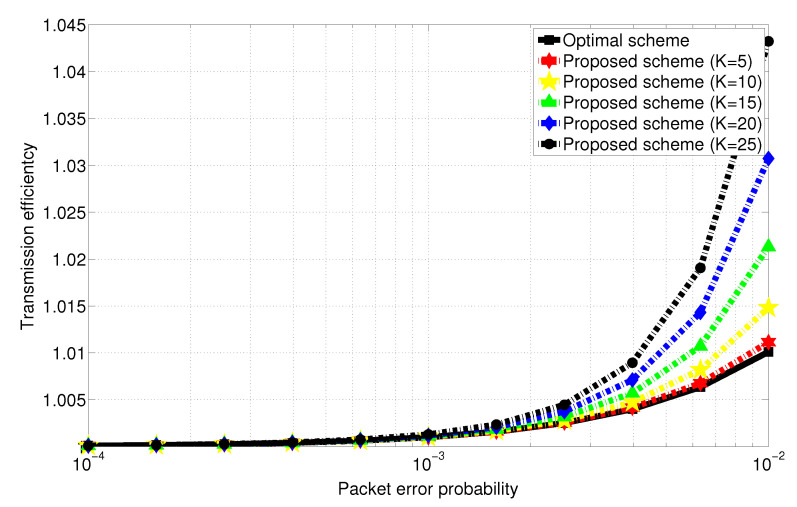
Transmission efficiency versus packet error probability with respect to *K* when 5≤K≤25. (The optimal scheme is also shown for comparison.)

Figure 1 depicts transmission efficiency defined as the total number of transmission normalized to *N*. As the number of Rxs increases, the proposed scheme shows lower transmission efficiency in comparison with the optimal scheme. However, the performance degradation is observed to be negligible as the packet error probability *P* goes to 0, which is confirmed from Figure 1. In other words, the transmission efficiency of the proposed scheme becomes prominent as the packet error probability *P* goes to 0 in the finite range of *K*. Furthermore, when *K* increase, the proposed scheme is observed to guarantee nearly optimal transmission efficiency from Equations (22) and (23).

### 6.2. Memory Overhead

**Figure 2:**
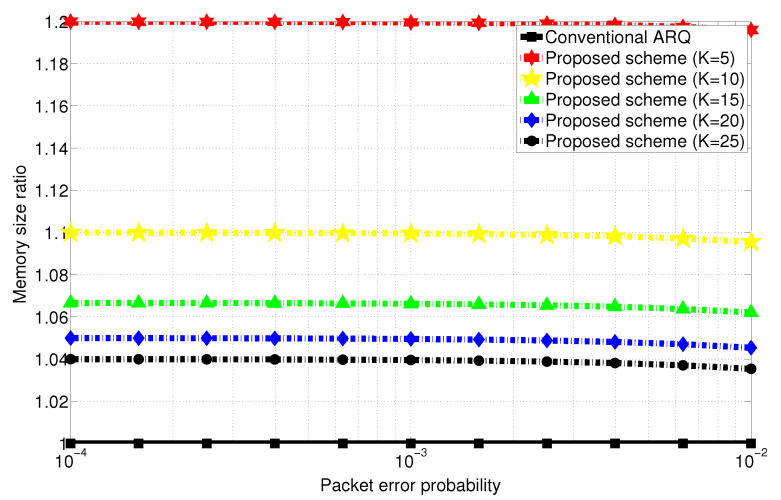
Memory size ratio versus packet error probability with respect to *K* when 5≤K≤25. (The conventional ARQ scheme is also shown for comparison.)

The proposed scheme, that is, index-coded ARQ requires additional memory for storing coded side information at the Tx and all Rx in comparison with the conventional ARQ. Basically, considering all combinations of index-coded packets can result in significant memory overhead as manifested in Equation (15). When the packet error probability *P* goes to 0, considering dominant packet error pattern can be sufficient to achieve appropriate tradeoff between transmission efficiency and memory overhead. Specifically, the packet error pattern set of $M^{\left[K-1\right]}$ is the most dominant, and the number of index coding combination is always 1 regardless of *K*. Consequently, it is observed that memory overhead is decreased as *K* increases interestingly, which is confirmed from Equations (16) and (17). Figure 2 depicts the memory size ratio of the proposed scheme with respect to the conventional ARQ scheme, which shows that the proposed scheme requires memory size overhead approximately within 10% when the number of users are greater than or equal to 10, whose phenomenon becomes distinct when *K* increases. Thus, the memory overhead of the proposed scheme is observed to be not significant, which supports the feasibility for practical use.

## 7. Conclusions

In the framework of packet transmission with subsequent retransmission, two contradictory performance metrics, that is, transmission efficiency and memory overhead are usually conflicting characteristics, which are difficult to be optimized simultaneously. In this paper, retransmission scheme was proposed considering those two contradictory performance metrics in a practical sense. Conventional ARQ scheme is one extreme in a sense that the transmission efficiency is relatively lower, but requires less computational complexity and moderate memory size overhead of O(N). The other extreme scheme corresponds to random linear network coding which achieves the optimal transmission efficiency at the cost of high computational complexity. In comparison with the two extremes, the proposed scheme was observed to achieve nearly optimal transmission efficiency, and the memory size overhead was not significant in comparison with the conventional ARQ scheme. Furthermore, the proposed scheme was shown to be asymptotically optimal in the non-negligible range of the packet error probability *P* with $P\leq O\left(K^{-(1+\epsilon )}\right)$ for any ϵ>0. By mathematical analysis and numerical results, the proposed scheme, that is, simplified index coded ARQ is expected to be a reasonable candidate for the efficient broadcast scheme for practical use.

## Figures and Tables

**Table 1:** Packet error pattern when K=2.

Packet error pattern set $\left(M^{\left[i\right]}\right)$	$M^{\left[0\right]}$	$M^{\left[1\right]}$		$M^{\left[2\right]}$
Packet error pattern subset $\left(M_{j}^{\left[i\right]}\right)$	$M_{1}^{\left[0\right]}$	$M_{1}^{\left[1\right]}$	$M_{2}^{\left[1\right]}$	$M_{1}^{\left[2\right]}$
1st Rx	NACK	ACK	NACK	ACK
2nd Rx	NACK	NACK	ACK	ACK

**Table 2:** Packet error pattern when K=3.

Packet error pattern set $\left(M^{\left[i\right]}\right)$	$M^{\left[0\right]}$	$M^{\left[1\right]}$			$M^{\left[2\right]}$			$M^{\left[3\right]}$
Packet error pattern subset $\left(M_{j}^{\left[i\right]}\right)$	$M_{1}^{\left[0\right]}$	$M_{1}^{\left[1\right]}$	$M_{2}^{\left[1\right]}$	$M_{3}^{\left[1\right]}$	$M_{1}^{\left[2\right]}$	$M_{2}^{\left[2\right]}$	$M_{3}^{\left[2\right]}$	$M_{1}^{\left[3\right]}$
1st Rx	NACK	ACK	NACK	NACK	ACK	ACK	NACK	ACK
2nd Rx	NACK	NACK	ACK	NACK	ACK	NACK	ACK	ACK
3rd Rx	NACK	NACK	NACK	ACK	NACK	ACK	ACK	ACK

**Table 3:** Transmission efficiency when K=2 and K=3.

The Number of Rxs	K=2			K=3		
Packet error probability	P = 0.001	P = 0.01	P = 0.1	P = 0.001	P = 0.01	P = 0.1
Optimal scheme (A)	1.0010	1.0101	1.1111	1.0010	1.0101	1.1111
Index coded ARQ (B)	1.0010	1.0102	1.1234	1.0010	1.0104	1.1402
(B−A)/A(%)	0.00	0.01	1.10	0.00	0.03	2.62
